# *TMEM196* acts as a novel functional tumour suppressor inactivated by DNA methylation and is a potential prognostic biomarker in lung cancer

**DOI:** 10.18632/oncotarget.4237

**Published:** 2015-05-22

**Authors:** Wen-bin Liu, Fei Han, Xiao Jiang, Hong-qiang Chen, Huan Zhao, Yong Liu, Yong-hong Li, Chuanshu Huang, Jia Cao, Jin-yi Liu

**Affiliations:** ^1^ Institute of Toxicology, College of Preventive Medicine, Third Military Medical University, Chongqing, P. R. China; ^2^ Department of Internal Neurology, Southwest Hospital, Third Military Medical University, Chongqing, P. R. China; ^3^ Nelson Institute of Environmental Medicine, New York University School of Medicine, Tuxedo, New York, USA

**Keywords:** TMEM196, tumour suppressor, lung cancer, DNA methylation, prognosis

## Abstract

Epigenetic silencing of tumour suppressors contributes to the development and progression of lung cancer. We recently found that *TMEM196* was hypermethylated in lung cancer. This study aimed to clarify its epigenetic regulation, possible roles and clinical significance. *TMEM196* methylation correlated with loss of protein expression in chemical-induced rat lung pathologic lesions and human lung cancer tissues and cell lines. 5-aza-2′-deoxycytidine restored *TMEM196* expression. Moreover, *TMEM196* hypermethylation was detected in 61.2% of primary lung tumours and found to be associated with poor differentiation and pathological stage of lung cancer. Functional studies showed that ectopic re-expression of *TMEM196* in lung cancer cells inhibited cell proliferation, clonogenicity, cell motility and tumour formation. However, *TMEM196* knockdown increased cell proliferation and inhibited apoptosis and cell-cycle arrest. These effects were associated with upregulation of *p21* and *Bax*, and downregulation of *cyclin D1*, *c-myc*, *CD44* and *β-catenin*. Kaplan–Meier survival curves showed that *TMEM196* downregulation was significantly associated with shortened survival in lung cancer patients. Multivariate analysis showed that patients with *TMEM196* expression had a better overall survival. Our results revealed for the first time that *TMEM196* acts as a novel functional tumour suppressor inactivated by DNA methylation and is an independent prognostic factor of lung cancer.

## INTRODUCTION

Lung cancer remains the most common cancer and first leading cause of cancer-related deaths worldwide [1]. Although several diagnostic techniques and treatments for lung cancer have been developed, the overall 5-year survival has not increased significantly because of poor prognosis and the lack of effective early detection methods [2]. To improve the survival rate of lung cancer patients, novel strategies for treating lung cancer need to be urgently pursued, especially molecularly targeted therapies. In addition, better understanding of the key molecular changes in normal cells that lead to precancerous lesions and malignant tumour cells will further the development of potential treatment for this disease.

The initiation and progression of lung cancer involves a multi-step process with sequential genetic and epigenetic changes [3-5]. Although the molecular mechanisms of lung carcinogenesis remain unclear, DNA methylation is the third most common mechanism of tumour suppressor gene inactivation and tumourigenesis, following the loss of heterozygosity and acquisition of mutations, and plays an important role in cancer development [6, 7]. DNA methylation patterns in tumourigenesis include both genome-wide hypomethylation and CpG islands hypermethylation, along with enhancement of total cellular methylation capacity. Its main significance may be the molecular basis for proto-oncogene activation, tumour suppressor gene inactivation and genomic instability [8-10]. Recent studies have demonstrated that a series of methylation-silenced tumour suppressor genes are associated with human cancer carcinogenesis, tumour progression and prognosis [11-19]. Thus, the characterisation of novel functional genes associated with CpG island methylation may help provide insights into the mechanisms for the inactivation of the tumour suppressive pathways involved in lung carcinogenesis and help identify better potential targets for the diagnosis and treatment of lung cancer.

Through genome-wide methylation screening, we identified a novel preferentially methylated gene, *transmembrane protein 196* (*TMEM196*), in human lung cancer [20], suggesting that it may be associated with lung tumourigenesis. However, few studies have investigated the regulation of *TMEM196*, and the role and function of *TMEM196* in lung cancer remain unknown. In the present study, we studied the promoter methylation and expression status of *TMEM196* in a chemical-induced rat lung cancer model, primary human tumour tissues and multiple lung cancer cell lines. We further investigated the biological functions, molecular basis and clinical significance of *TMEM196* in lung cancer.

## RESULTS

### *TMEM196* is hypermethylated in chemical-induced rat lung lesions, human lung cancer tissues and cell lines

**Figure 1:**
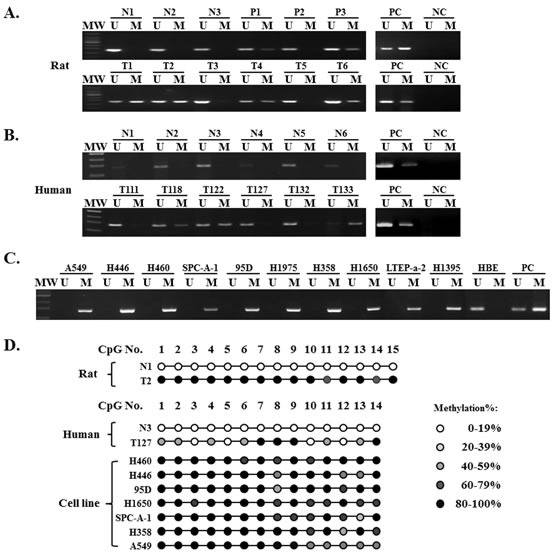
Representative results of methylation analysis of *TMEM196* promoter CpG islands in a chemical-induced rat lung cancer model and human tissue and cell line samples by MSP and BGS **A.** *TMEM196* gene was unmethylated in all rat normal tissues and methylated in precancerous and tumour tissues. MW: molecular weight; U: PCR product amplified by unmethylated-specific primers; M: PCR product amplified by methylated-specific primers; N: normal tissue; P: precancerous tissues; T: tumour tissues; PC: positive control, including fully unmethylated control and fully methylated control; NC: negative control. **B.** *TMEM196* gene was unmethylated in all human normal tissues and methylated in primary human tumour tissues. **C.** *TMEM196* gene was fully methylated in all human lung cancer cell lines, but unmethylated in the normal HBE cell line. **D.** Representative BGS analysis of *TMEM196* promoter methylation in rat and human tissues and cell line samples.

First, we used methylation-specific polymerase chain reaction (MSP) to examine the methylation state of the *TMEM196* gene in chemical-induced lung carcinogenesis in rats (Figure 1A). Primer information was shown in Supplementary Table S1. Unmethylated *TMEM196* alleles were only detected in normal epithelium and hyperplasia. CpG methylation of *TMEM196* was detectable in various precancerous and tumour cells after laser capture microdissection. The frequency of *TMEM196* methylation correlated with the pathological severity of lung carcinogenesis, with a gradual increase in methylation frequency from 14.8% (4/27) in squamous metaplasia, 29.7% (11/37) in dysplasia, 40.0% (12/30) in carcinoma in situ (CIS), and finally 52.0% (13/25) in infiltrating carcinoma samples (Supplementary Table S2).

Next, we evaluated *TMEM196* methylation status in 85 cases of human primary lung cancer and 20 cases of normal lung tissue. Using MSP, we found *TMEM196* hypermethylation in 52 out of 85 (61.2%) lung cancer samples compared with no methylation in all examined normal lung tissues (0/20) (representative results shown in Figure 1B).

Next, we analysed the association of *TMEM196* methylation status and clinicopathological characteristics in 62 lung cancer patients with all parameters available. As shown in Supplementary Table S3, there was no correlation between *TMEM196* hypermethylation and clinicopathological features such as age, gender, smoking, or histological type. However, *TMEM196* methylation was associated with poor differentiation (*P =* 0.039) and pathological stage (*P* = 0.017) of lung cancer. We then evaluated the methylation status of *TMEM196* in several human lung cancer cell lines using MSP. The results showed that all 10 lung cancer cell lines in our study show *TMEM196* hypermethylation, while the normal human bronchial epithelial cell line HBE exhibited unmethylation status (Figure 1C).

To provide a detailed map of the DNA methylation pattern within the CpG island region of the *TMEM196* gene (Supplementary Figure S1), we performed bisulphite genomic sequencing (BGS) (representative results shown in Figure 1D). BGS results were in good agreement with the MSP findings, with *TMEM196* being densely methylated at the promoter in most of the cell lines, partially methylated in tumour tissues and unmethylated in normal tissues.

### *TMEM196* downregulation or inactivation is associated with DNA methylation in rat and human primary lung cancer tissues and cell lines

**Figure 2:**
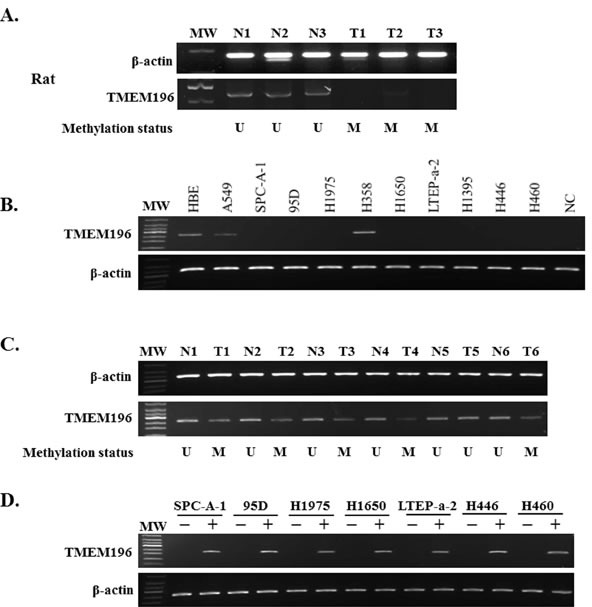
Epigenetic inactivation of *TMEM196* in a rat cancer model and human tissues and cell lines **A.** Methylation of the *TMEM196* promoter correlated with the loss of mRNA expression in chemical-induced lung pathologic lesions. U: unmethylation; M: methylation. **B.** *TMEM196* transcript was reduced or silenced in nine cell lines but was readily detected in H358 cells and the normal cell line HBE. **C.** *TMEM196* expression was downregulated in lung cancer tissues (T) with hypermethylation status compared with their adjacent normal tissues (N) with unmethylation status. **D.** Re-expression of *TMEM196* in SPC-A-1, 95D, H1975, H1650, LTEP-a-2, H446 and H460 cell lines with low levels of expression and full methylated promoter region by pharmacologic demethylation. −: DMSO control; +: 5-aza-dC.

To determine the relationship between hypermethylation of the *TMEM196* gene and its expression, we examined TMEM196 expression in the chemical-induced rat model. We found that TMEM196 expression was decreased in chemical-induced, rat lung pathologic lesions (Figure 2A and Supplementary Table S2). We next performed a correlation analysis between *TMEM196* promoter methylation and expression shown in Supplementary Table S4. There was concordance between the methylation status and protein expression for TMEM196 in all but 12 samples. The 112 samples with unmethylated *TMEM196* exhibited positive protein expression, while the 40 samples with methylated *TMEM196* were negative for protein expression. There was a statistically significant negative correlation between *TMEM196* promoter methylation and its protein expression during chemically induced rat lung carcinogenesis, especially in the stages of squamous metaplasia, dysplasia, CIS and infiltrating carcinoma (*P* < 0.01, Supplementary Table S4).

To clarify whether DNA hypermethylation regulated the expression of *TMEM196* gene, we examined *TMEM196* mRNA expression in 10 human lung cancer cell lines and the normal HBE cell line by reverse transcription-polymerase chain reaction (RT-PCR) and quantitative RT-PCR. As shown in Figure 2B and Supplementary Figure S2, the *TMEM196* transcript was reduced or silenced in nine (90%) cell lines but was readily detected in H358 cells and the normal cell line HBE. *TMEM196* expression level was inversely correlated with the methylation status in all lung cancer cell lines, except H358 cells, and HBE cells. We next evaluated *TMEM196* mRNA expression in 10 primary lung cancers and their corresponding adjacent non-tumour tissues by RT-PCR. As shown in Figure 2C, *TMEM196* expression was downregulated in lung cancer tissues compared with their adjacent normal tissues. These results suggest that *TMEM196* hypermethylation was associated with its transcriptional downregulation or silencing.

To further determine whether *TMEM196* expression was regulated by promoter region methylation, we used the demethylating agent 5-aza-2′-deoxycytidine (5-aza-dC). The representative results of RT-PCR analyses were shown in Figure 2D. As expected, re-expression of *TMEM196* was induced in SPC-A-1, 95D, H1975, H1650, LTEP-a-2, H446 and H460 cell lines with low levels of expression and a full methylated promoter region. These results further indicate that *TMEM196* expression is regulated by promoter methylation.

### Tumour-suppressive function of *TMEM196*

**Figure 3:**
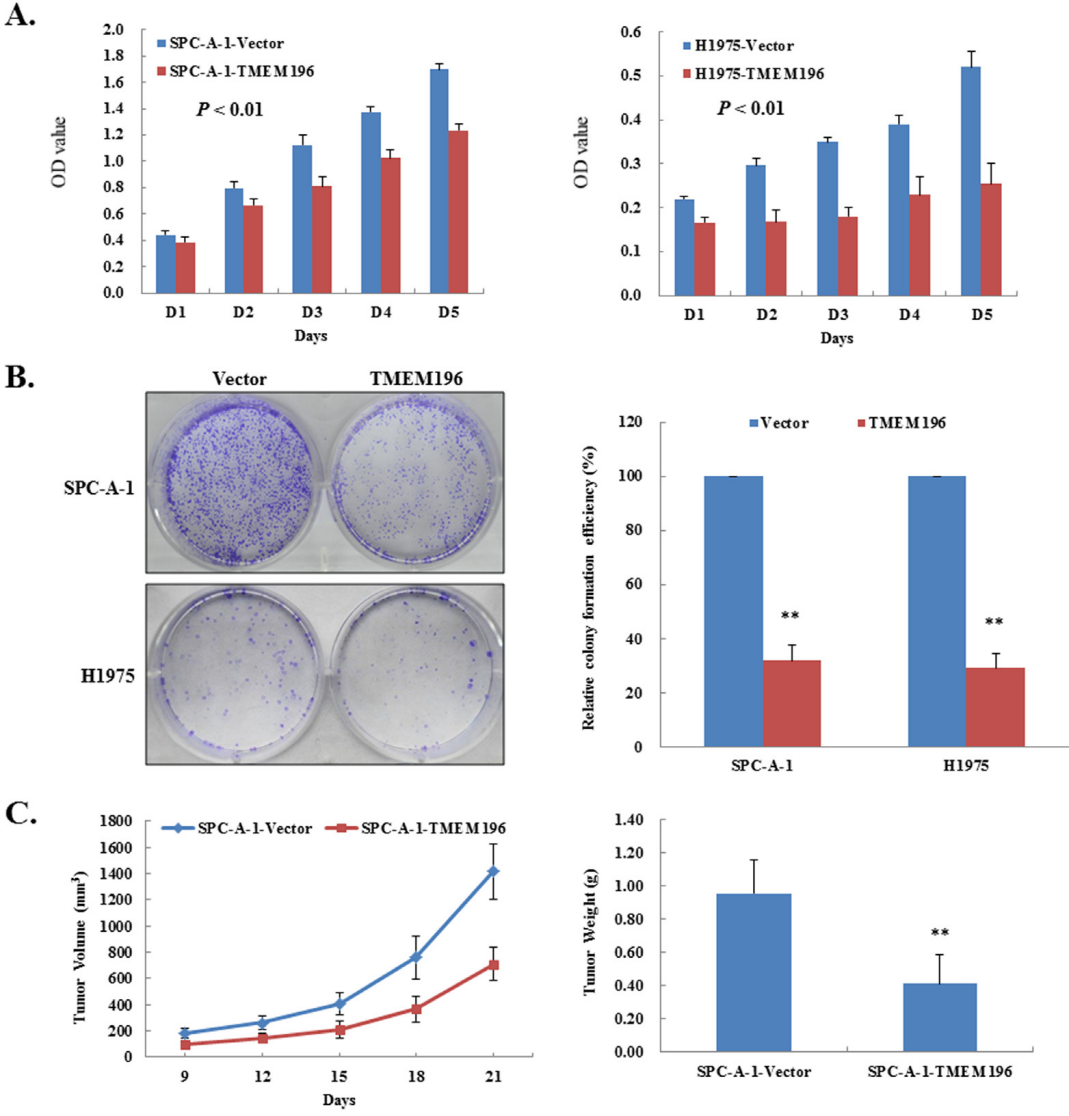
*TMEM196* suppressed lung cancer cell growth **A.** Overexpression of *TMEM196* inhibited cancer cell proliferation measured by CCK-8 assay. The values obtained from transfected and control cells represent mean ± SD of three independent experiments. **B.** Representative colony formation assays. Quantitative analyses of colony numbers are shown as values of mean ± SD (right panel). **C.** Inhibition of tumour growth by *TMEM196* expression *in vivo*. The average size and weight of tumours induced by *TMEM196*-expressing cells was significantly smaller than the control. **P < 0.01.

To characterise its tumour suppressive function, we transfected the *TMEM196* gene into SPC-A-1 and H1975 cell lines with methylated and silenced *TMEM196*. Expression of *TMEM196* was confirmed by EGFP observation and RT-PCR (Supplementary Figure S3). Cell counting assay found that *TMEM196* could induce a significant time-dependent inhibition of cell proliferation in SPC-A-1 and H1975 cell lines (Figure 3A). Similarly, colony formation assay showed that *TMEM196* significantly suppressed colony formation (colony numbers down to ~20–40% of controls) in both tested cell lines compared with vector control cells (Figure 3B). The tumour suppressing effects of *TMEM196* was demonstrated in H358 cells with normal *TMEM196* expression levels (Supplementary Figure S4).

To further determine the tumour suppressive ability of *TMEM196 in vivo*, we evaluated tumour formation in nude mice. We found that the average size and weight of tumours induced by *TMEM196*-expressing cells significantly decreased compared with the controls (*P* < 0.01; Figure 3C and Supplementary Figure S5). These data showed that *TMEM196* indeed had tumour-suppressive ability both *in vivo* and *in vitro* in lung cancer.

### *TMEM196* arrests cell cycle and induces cell apoptosis

**Figure 4:**
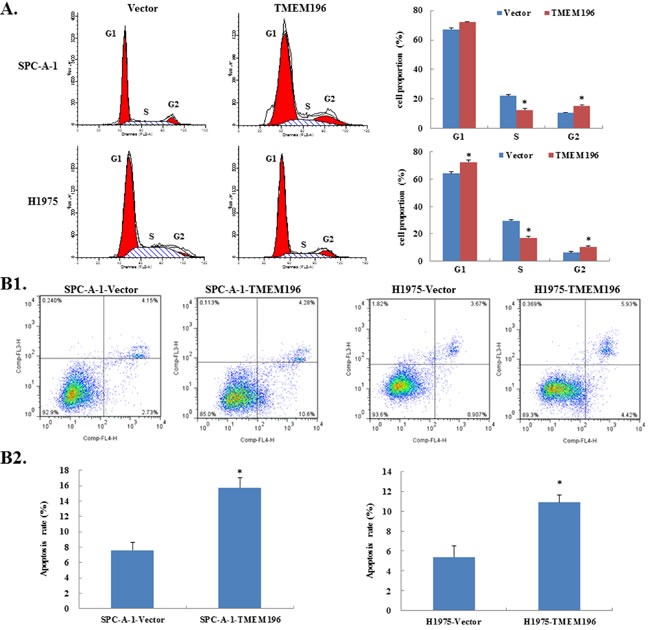
*TMEM196* induced cell-cycle arrest and apoptosis **A.** Cell-cycle profiles in cancer cells after *TMEM196* overexpression were determined by flow cytometry. Representative cell-cycle analysis and summarized flow cytometry data are shown. Results are represented as mean ± SD and based on three independent experiments. **B.** *TMEM196* induced apoptosis in SPC-A-1 and H1975 cells as shown by flow cytometric analysis. (B1) Representative dot plots of lung cancer cells transfected with empty vector or *TMEM196* by double staining with Annexin-V-APC and 7-AAD staining kit and flow cytometry. (B2) Quantitative analyses showed that the percentage of apoptotic cells was significantly increased in *TMEM196* transfectants compared with empty vector-transfected cells. *P < 0.05. The experiment was repeated in triplicate. Data are the mean ± SD, *P < 0.05.

To clarify the molecular mechanism of the inhibition effect of tumour cell growth by *TMEM196*, we examined the cell cycle and apoptosis by flow cytometry. SPC-A-1 cells overexpressing *TMEM196* showed a cell-cycle arrest at the G2/M checkpoint, with an accumulation of cells in G2 phase and a decrease in S-phase cells compared with control cells (G2 phase: 15.28±0.82% *vs*. 10.76±0.30%, respectively; S-phase: 12.51±1.17% *vs*. 22.14±0.83%) (Figure 4A). In addition, we also found a slight increase in G1 phase cells in *TMEM196*-reexpression cells, but the increase was not significant. In H1975 cells, overexpression of *TMEM196* significantly increased cells at G1 phase (72.37±1.68% *vs*. 64.14±1.16%) and G2 phase (10.53±0.64% *vs*. 6.14±0.72%), and decreased cells in S phase (17.11±1.04% *vs*. 29.72±0.44%) compared with control cells (Figure 4A).

Next, we used Annexin V-APC/7-AAD Apoptosis Detection kit to examine apoptosis (Figure 4B1). We found that the percentage of apoptotic cells was significantly higher in *TMEM196* transfectants compared with empty vector-transfected cells (SPC-A-1: 15.77±1.26% *vs*. 7.62±1.05%, respectively; H1975: 10.89±0.76% *vs*. 5.39±1.15%) (Figure 4B2).

### *TMEM196* suppresses cell migration

**Figure 5:**
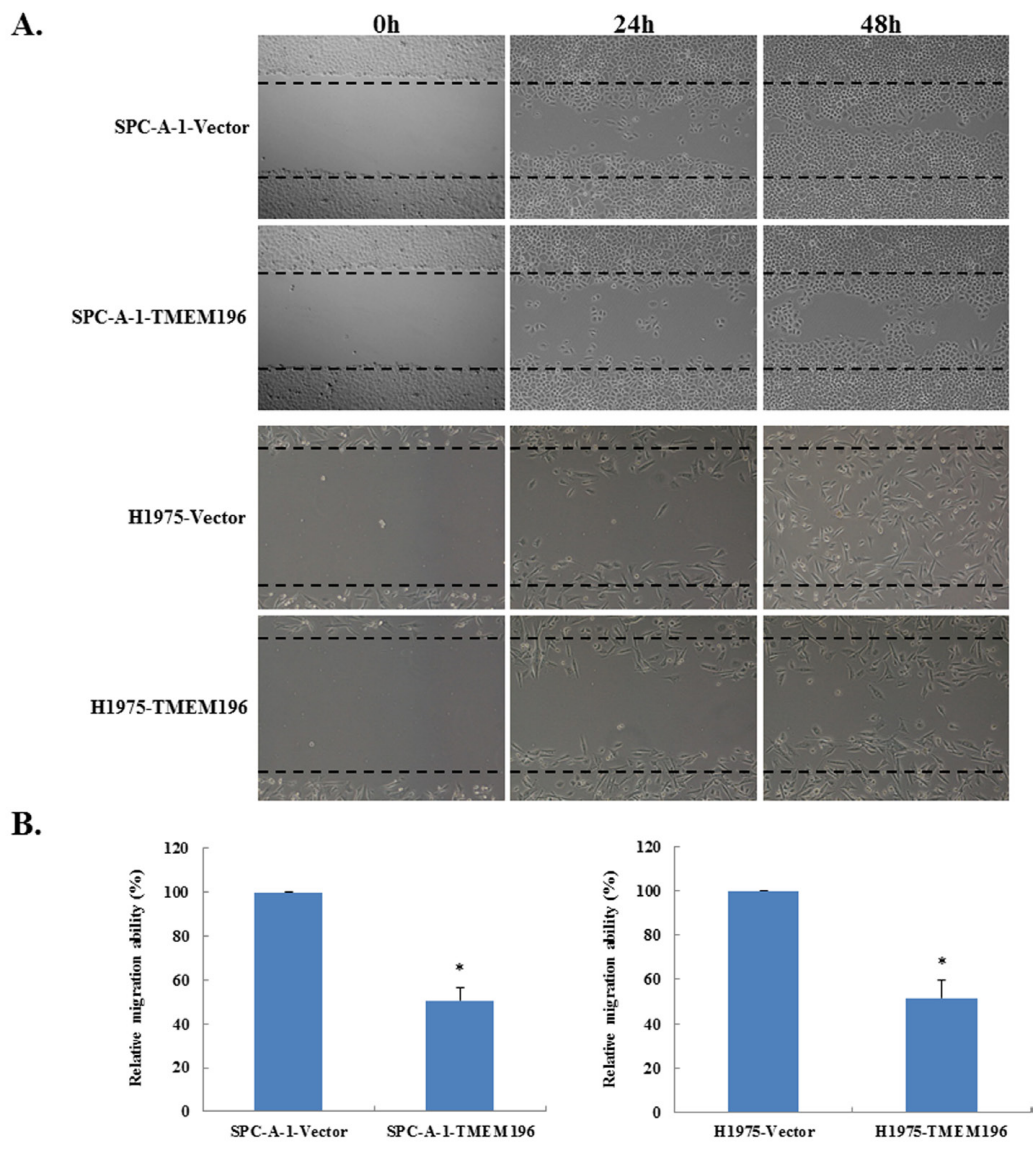
*TMEM196* inhibits cell migration **A.** Cell migration of *TMEM196*- or empty vector-transfected SPC-A-1 and H1975 cells was analysed by a monolayer scratch assay. Magnified area by phase-contrast microscopy 24 h and 48 h after scratching. **B.** Detailed quantification comparison of wound closure after 48 h. *P < 0.05.

We used the wound healing assay to detect the effect of *TMEM196* on lung cancer cell motility. Confluent monolayers of vector- and *TMEM196*-transfected SPC-A-1 and H1975 tumour cells were scratched 48 h after transfection. Phase contrast microscopy photos of wound margins were taken at 24 and 48 h after scratching. Results showed that *TMEM196* transfectants spread along the wound edges much slower than control vector transfectants (Figure 5A). Quantitative analyses at 48 h confirmed that wound closure was significantly decreased in *TMEM196*-transfected cells compared with empty vector-transfected control cells (Figure 5B).

### Knockdown of *TMEM196* promotes cell proliferation

**Figure 6:**
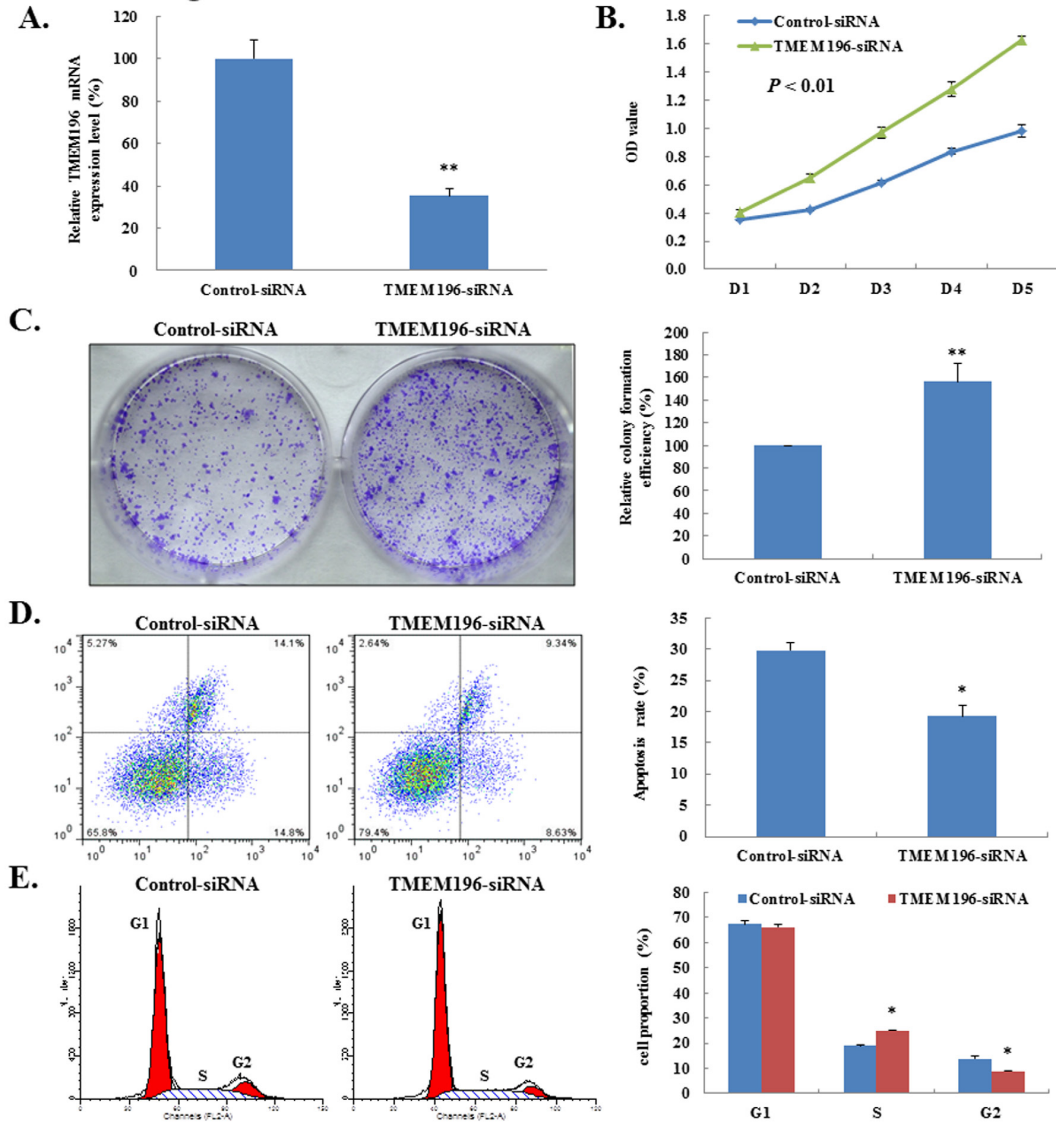
Knockdown of *TMEM196* promotes cell growth **A.** *TMEM196* gene knockdown mediated by siRNA was examined by real-time RT-PCR analysis in HBE cells. **B.** Knockdown of *TMEM196* promotes cell proliferation as determined by CCK-8. **C.** *TMEM196* knockdown caused a significant increase in cell colony formation in HBE cells. The colonies photographed under a phase-contrast microscope are shown on the left, and the quantification of average number of tumour clones by bar diagram is shown on the right. **D.** Knockdown of *TMEM196* expression significantly reduced apoptosis of HBE cells as determined by flow cytometry. **E.** *TMEM196* expression knockdown led to a significant decrease in the number of HBE cells in G2 phase. Each experiment was repeated three times. *P < 0.05; **P < 0.01.

To further confirm the tumour suppressor role of *TMEM196* in lung cancer, we used siRNA vector transfection to knockdown *TMEM196* expression in the *TMEM196*-expressing cell line HBE (Supplementary Figure S6). Figure 6A shows that *TMEM196* was significantly decreased by more than 60% in *TMEM196*-siRNA transfectants compared with control cells by real-time quantitative RT-PCR. RNAi-mediated knockdown of endogenous *TMEM196* significantly enhanced the growth of HBE cells by ~30% (*P* < 0.01, Figure 6B). Colony formation assay further confirmed this knockdown of *TMEM196* resulted in significantly increased colony formation ability (by more than 60%) compared with the siRNA vector control (Figure 6C). These data further suggest that *TMEM196* acts as a potential tumour suppressor through inhibiting cell growth in lung cancer.

Next, we examined the effect of knockdown of *TMEM196* expression on apoptosis and cell-cycle regulation. The apoptotic cells in *TMEM196* siRNA-transfected HBE cells decreased significantly compared with cells transfected with control siRNA vector (19.25±1.80% vs. 29.80±7.27%, respectively; *P* < 0.05) (Figure 6D). Cell-cycle analysis showed that transfection with *TMEM196* siRNA decreased the fraction of cells in G2 phase (Figure 6E), suggesting that loss of *TMEM196* expression promotes cellular proliferation.

### Downstream genes of *TMEM196* in cell lines

**Figure 7:**
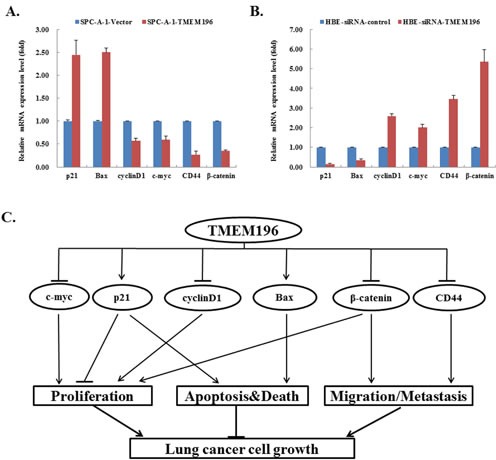
Downstream genes of *TMEM196* in lung cancer **A.** The expression of *p21* and *Bax* increased, while the expressions of *cyclinD1*, *c-myc*, *CD44* and *β-catenin* were downregulated in SPC-A-1 cells with *TMEM196* overexpression. **B.** Expression of *p21* and *Bax* decreased, while the expression of *cyclinD1*, *c-myc*, *CD44* and *β-catenin* increased in HBE cells with knockdown of *TMEM196*. **C.** Schematic for the molecular mechanisms of *TMEM196* as a tumour suppressor.

To investigate the possible downstream genes modulated by *TMEM196*, we evaluated several important genes in cell proliferation, apoptosis and migration by quantitative RT-PCR analysis. In SPC-A-1 cells stably transfected with *TMEM196*, expressions of the pro-apoptotic gene *Bax* and cell-cycle regulator *p21* were increased, while the expressions of cell proliferation genes *cyclin D1* and *c-myc* and cell adhesion-related genes *CD44* and *β-catenin* were decreased compared with the vector group (Figure 7A). However, knockdown of *TMEM196* expression downregulated the expression of *p21* and *Bax*, and enhanced *cyclin D1*, *c-myc*, *CD44* and *β-catenin* in HBE cells (Figure 7B). These results suggest that *TMEM196* regulates the expression of these important genes associated with cell proliferation, apoptosis and migration pathways (Figure 7C).

### Low TMEM196 expression is associated with poor survival of lung cancer patients

**Figure 8:**
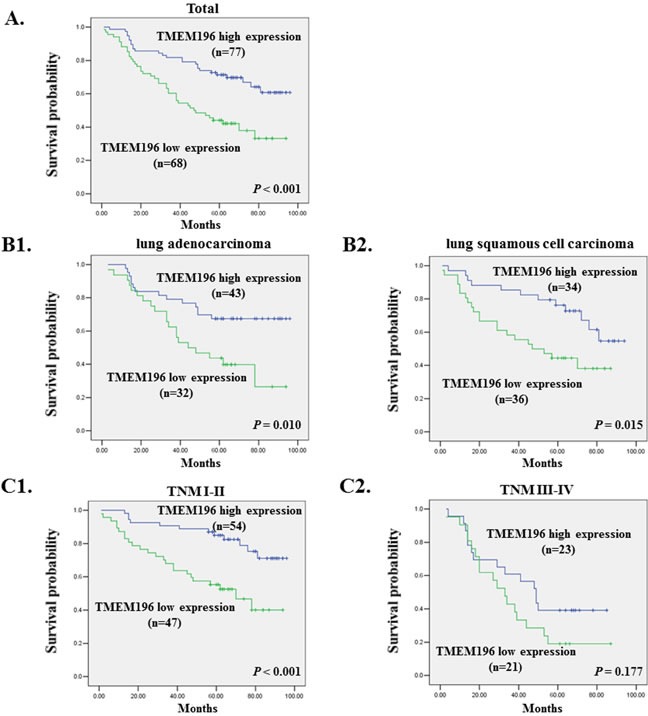
Low TMEM196 expression is associated with poor survival of lung cancer patients **A.** Kaplan–Meier survival curves show that lung cancer patients with low TMEM196 expression had poorer survival than those with high TMEM196 expression based on a log-rank test (*P* < 0.001). **B.** Kaplan–Meier curves of lung cancer patients stratified by expression status of different tumour types including adenocarcinoma (B1) and squamous cell carcinoma (B2) showed that survival was significantly shorter in the low TMEM196 expression group. **C.** Kaplan–Meier curves of lung cancer patients in tumour-nodes-metastasis (TNM) stages I–II (C1) and TNM stages III–IV (C2).

To evaluate the clinical significance of TMEM196, immunohistochemical analysis of tissue microarray (TMA) of 145 lung cancer patients was performed. We found that TMEM196 was expressed at low levels in lung cancer tumour tissues and expressed at high levels in adjacent tissue sample (Supplementary Figure S7). Kaplan–Meier survival curves showed that lung cancer patients with low TMEM196 expression had significantly shorter survival than those with high TMEM196 expression (*P* < 0.001, log-rank test; Figure 8A). Based on Kaplan–Meier curves of lung cancer patients stratified by expression status of different tumour type including adenocarcinoma (Figure 8B1) and squamous cell carcinoma (Figure 8B2), survival was significantly shorter in the low expression group. In addition, patients with low TMEM196 expression in TNM stages I–II showed a significantly poorer survival than patients with high TMEM196 expression (*P* < 0.001; Figure 8C1), but patients in TNM stages III–IV did not differ significantly (*P =* 0.177; Figure 8C2).

The characteristics of patients with lung cancer associated with the survival status are shown in Table 1. In the univariate Cox regression analysis, TMEM196 expression in tumour tissues was associated with a decreased risk of cancer-related death (hazard ratio (HR) = 0.456; 95% confidence interval (CI), 0.253–0.822; *P =* 0.009). After adjustment for potential confounding factors, TMEM196 expression in tumour tissues was found to predict better survival (HR = 0.357; 95% CI, 0.204–0.624; *P* = 0.0003) in the multivariate model (Table 1). This suggests that TMEM196 expression was an independent predictor of poorer survival of patients with lung cancer. As expected, tumour stage was another independent predictor for overall survival. Patients in stage I had a significantly better survival compared with patients with a stage IV tumour (Table 1).

**Table 1:** Multivariate Cox regression analysis of potential prognostic factors for lung cancer patients

Vleariab	HR (95% CI)	*P* value
**Age(year)**	1.046 (1.016-1.077)	0.002
**Gender**		
female	1.00	
male	0.990 (0.474-2.070)	0.979
**Grade**	1.149 (0.650-2.034)	0.632
**Tumor size**	1.033 (0.863-1.236)	0.726
**Total number of lymph nodes**	0.949 (0.903-0.997)	0.037
**Positive number of lymph nodes**	1.010 (0.879-1.160)	0.890
**Stage**		
I	1.00	
II	2.416 (1.198-4.871)	0.014
III	5.380 (2.708-10.692)	<0.0001
IV	7.690 (2.034-29.081)	0.003
**TMEM196 expression**		
Low	1.00	
High	0.357 (0.204-0.624)	0.0003

Abbreviations: HR, hazard ratio; CI, confidence interval.

## DISCUSSION

DNA methylation is an important mechanism in the downregulation and silencing of tumour suppressor genes in tumour initiation and progression [21-27]. Using genome-wide methylation screening, we identified *TMEM196* hypermethylation in lung cancer. To the best of our knowledge, this is the first study on the epigenetic regulation of *TMEM196* and its function in lung cancer.

In this study, we found that *TMEM196* was hypermethylated in human primary lung cancer tissues and cell lines but not in corresponding normal tissues. Furthermore, we showed that the *TMEM196* gene is frequently methylated in the chemical-induced rat lung cancer model, and the methylation frequency correlated with the lung pathologic lesions and loss of expression of the protein. These data showed that *TMEM196* hypermethylation is an early event in the development of lung carcinogenesis. In the future, the sputum and plasma data should be validated in a large prospective screening study for lung cancer, and *TMEM196* methylation should be combined with other methylation markers to enhance the sensitivity and/or specificity.

*TMEM196*, located on human chromosome 7p21.1, encodes a transmembrane protein containing 172 amino acids and four transmembrane regions [28]. TMEM196 belongs to the transmembrane protein family, which plays important roles in basic physiological processes, including differentiation, migration, adhesion, aggregation, dissolving and signal transduction regulation in diseases [29-32]. Previous studies found that *HPP1*, *VEZT* and *IFITM3*, which contain a similar transmembrane domain to that of TMEM196, were hypermethylated associated with downregulation in colorectal cancer, gastric cancer and melanoma, respectively [33-35]. Recently, several other transmembrane proteins have also been implicated in tumourigenesis [36-43]. TMEM16A inhibits tumour growth both *in vitro* and *in vivo* through the ERK/MAPK pathway [36]. TMEM34 downregulates and inhibits cell growth in anaplastic thyroid cancer [37]. TMEM147 stimulates cell proliferation in colorectal cancer through negative regulation of the M_3_ muscarinic receptor expression [38]. TMEM166 inhibits human cancer cell growth by autophagy and apoptosis *in vitro* and *in vivo* [39, 40], and exhibits the same effects following focal cerebral ischemic injury in rats [41]. TMEM207 binding with the WW domain-containing oxidoreductase promotes cell migration and invasion in gastric cancer [42]. TMEM214 mediates endoplasmic reticulum stress-induced caspase-4 activation and apoptosis [43]. These results suggest that *TMEM196* expression may be associated with lung carcinogenesis. In addition, several novel genes inactivated by DNA methylation have been found to suppress cell growth in several types of tumours [14-19]. From our findings, we propose the hypothesis that *TMEM196* expression may function as a potential tumour suppressor in lung cancer.

To explore the tumour suppressive function of *TMEM196* in lung cancer, functional analysis of *TMEM196* was performed *in vitro* and *in vivo*. We found that ectopic expression of *TMEM196* in lung cancer cell lines SPC-A-1 and H1975 could inhibit cell growth, colony formation and tumour formation in nude mice. Furthermore, knockdown of *TMEM196* mediated by siRNA vector significantly promoted cell proliferation in HBE cells. These results suggested that *TMEM196* functions as a tumour suppressor in lung cancer. Further studies showed that *TMEM196* could induce cell-cycle arrest and inhibit cell migration. Molecular studies revealed that the tumour suppressive role of *TMEM196* was closely associated with its pro-apoptotic effect.

We further evaluate the clinical significance and prognostic value of TMEM196 in lung cancer. We found that TMEM196 expression in tumour tissues was found to predict better survival in the multivariate model. These results indicate that TMEM196 expression could be an independent prognostic marker for lung cancer patients. As the TNM stage is a highly important predictor of disease recurrence, we used Kaplan–Meier curves stratified by both expression status and TNM stage. The results showed that low TMEM196 expression was significantly associated with shorter survival for TNM stage I–II lung cancer patients. These data suggest that TMEM196 expression may specifically predict the most aggressive and fatal types of lung cancer at an early stage.

In summary, our study identified *TMEM196* as a functional tumour suppressor with frequent epigenetic inactivation in lung cancer. The methylation-mediated silencing of *TMEM196* may serve as a potential epigenetic biomarker for early diagnosis and a therapeutic target for patients with lung cancer. However, further investigations are needed to delineate the exact *TMEM196* signalling pathways.

## MATERIALS AND METHODS

### Cell lines, tumours and normal tissue samples

Lung cancer cell lines (A549, SPC-A-1, 95D, H1975, H358, H1650, LTEP-a-2, H1395, H446 and H460) and the immortalised human bronchial epithelial cell line HBE were obtained from the American Type Culture Collection (Manassas, VA, USA) and the Cell Biology Institute of Chinese Academy of Science (Shanghai, China). All cell lines were cultured in RPMI-1640 medium (Gibco BRL, Rockville, MD, USA) with 10% fetal bovine serum (Gibco BRL) in a humidified atmosphere with 5% CO_2_ at 37°C.

Normal and different morphological rat multistep carcinogenesis tissues (hyperplasia, squamous metaplasia, dysplasia, CIS and infiltrating carcinoma) were obtained from a chemical-induced, rat lung cancer model in our laboratory as reported previously [21-25].

A total of 85 primary lung cancer tissues obtained at the Affiliated Xi'nan Hospital of the Third Military Medical University were investigated. Patients who received pre-operative chemotherapy were excluded. Twenty normal lung biopsy samples, obtained from healthy volunteers who underwent bronchoscopy for routine screening, were used as normal controls. Ten paired biopsy tissues from primary lung cancer and adjacent normal tissues were obtained at the Affiliated Xi'nan Hospital of the Third Military Medical University as previously reported [12]. All experiments and procedures were approved by the Clinical Research Ethics Committee of the Third Military Medical University.

### DNA extraction, MSP and BGS

Genomic DNA was isolated and chemically modified with the EZ DNA Methylation-Gold Kit (Zymo Research, Orange, CA, USA) according to the manufacturer's instruction. MSP and BGS were carried out as described previously [12, 13]. Primer pairs of rat and human *TMEM196* used for MSP and BGS were designed with Methprimer and are listed in Supplementary Table S1.

### RNA isolation, RT-PCR and real-time quantitative RT-PCR analyses

RNA isolation was performed by Trizol (Invitrogen, Carlsbad, CA, USA). The PrimeScript^®^ RT reagent Kit with gDNA Eraser (Takara, Otsu, Japan) was used to synthesise cDNA. RT-PCR and real-time quantitative RT-PCR analyses were performed as described previously with β-actin as an internal control [26, 27]. Primer information is listed in Supplementary Table 1.

### 5-aza-dC treatment

Briefly, cells were seeded at a density of 10^6^ cells/mL in 10-cm dishes for 24 h, and then incubated in fresh culture medium with or without the DNA demethylating agent 5-aza-dC (Sigma, St Louis, MO, USA) at a final concentration of 10 μM for 3 days. The medium and the drug were replaced every day. Cells were harvested and mRNA expression of *TMEM196* was analysed by RT-PCR.

### Generation of cell lines stably *TMEM196* overexpression and knockdown

The full-length human *TMEM196* gene cDNA was confirmed by sequencing and subcloned into the mammalian expression vector pIRES2-EGFP (Invitrogen, Carlsbad, CA, USA). Cells were transfected with the *TMEM196* vector or empty vector using the X-treme Gene HP DNA transfection reagent (Roche, Mannheim, Germany). Overexpression of *TMEM196* was confirmed by RT-PCR.

*TMEM196* mRNA siRNAs and the negative control sequence were designed, synthesised, and subcloned into pcDNA6.2™ GW/EmGFP siRNA vectors (Invitrogen). The HBE cell line with *TMEM196* expression was transfected with vectors carrying siRNA-*TMEM196* or the siRNA negative control. RNA was extracted 48 h after transfection and the siRNA producing the greatest *TMEM196* knockdown was used to assess cell function.

### Cell viability assay and colony formation assay

Briefly, cells (8×10^3^ per well) were seeded in 96-well plates and transfected with vectors. After 1–5 days of transfection, cell viability was determined using the Cell Counting Kit-8 (Dojindo, Kumamoto, Japan) according to the manufacturer's instructions. Experiments were carried out in triplicate.

After 48h of transfection, cells were cultured with G418 (0.4 mg/mL; Invitrogen) or Blasticidin S HCl (0.6 mg/mL; Invitrogen). Colony formation was analysed 14–21 days later by staining cells with crystal violet solution. Colonies with more than 50 cells per colony were counted. All experiments were conducted in triplicate.

### Cell cycle and apoptosis analysis

Cell-cycle profiles were determined using the ModFitLT software (Becton Dickinson, San Diego, CA, USA). Apoptosis was determined by Annexin V-APC/7-AAD Apoptosis Detection kit (Keygen, Nanjing, China) according to the manufacturer's instructions, and then analysed by FlowJo software (TreeStar, San Carlos, CA, USA).

### Wound-healing assay

Cell migration was assessed by a scratch wound assay. Briefly, cells (5×10^5^ cells/well) stably transfected with pIRES2-EGFP-*TMEM196* or empty vector were selected using G418 and then cultured in six-well plates until confluent. After scratching the monolayer, cells were photographed at 0, 24, and 48 h under a 10× objective (Olympus, Japan). Images were taken of six random optical fields (100×) on each filter. The experiments were conducted in duplicate.

### *In vivo* mouse models

Tumourigenicity in nude mice was determined as described previously [12, 13]. All experimental procedures were approved by the Animal Ethics Committee of the Third Military Medical University. SPC-A-1 cells (5×10^6^ cells in 0.2 mL PBS) stably transfected with pIRES2-EGFP-*TMEM196* or empty vector were injected subcutaneously into the right dorsal flank of 4-week-old female BALB/c nude mice (six mice/group). Tumour volume was calculated using the following formula: (short diameter)^2^×(long diameter)/2. Tumour volume was assessed every 3 days for 3 weeks.

### TMA and immunohistochemistry

Two TMA chips containing a total of 145 primary lung cancer tissues were obtained from Shanghai Biochip Company Ltd of China. Immunohistochemical staining for TMEM196 (sc-248960; Santa Cruz Biotechnology, Santa Cruz, CA, USA) was performed as described previously [21]. The intensity of staining was graded semi-quantitatively as negative (scored as 0), weak (1), moderate (2) or strong (3) positivity. The percentage of positive cells, as the extent of immunostaining, was quantified into five groups under microscope: < 10% positive cells for 0; 10–25% positive cells for 1; 26–50% positive cells for 2; 51–75% positive cells for 3 and ≥ 76% positive cells for 4. Multiplying the percentage of positive staining and the intensity was used to define expression levels. A final staining score > 4 was considered to be high expression.

### Statistical analysis

Results are expressed as values of mean ± standard deviation (SD). Results were evaluated using the *t*-test, Fisher exact test, and Mann–Whitney *U* test. Overall survival in relation to expression status was evaluated by the Kaplan–Meier survival curve and the log-rank test. HR of death associated with *TMEM196* expression and other predictor variables were estimated by Cox regression analysis. Statistical analysis was performed using SPSS 13.0 for Windows (SPSS Inc., Chicago, IL, USA). For all tests, *P* < 0.05 was considered of statistical significance.

## SUPPLEMENTARY MATERIAL FIGURES AND TABLES



## References

[1] Siegel R, Naishadham D, Jemal A (2013). Cancer statistics, 2013. CA Cancer J Clin.

[2] Hassanein M, Callison JC, Callaway-Lane C, Aldrich MC, Grogan EL, Massion PP (2012). The state of molecular biomarkers for the early detection of lung cancer. Cancer Prevention Research.

[3] Breuer RH, Pasic A, Smit EF, van Vliet E, Vonk Noordegraaf A, Risse EJ, Postmus PE, Sutedja TG (2005). The natural course of preneoplastic lesions in bronchial epithelium. Clin Cancer Res.

[4] Wu X, Wang L, Ye Y, Aakre JA, Pu X, Chang GC, Yang PC, Roth JA, Marks RS, Lippman SM, Chang JY, Lu C, Deschamps C (2013). Genome-wide association study of genetic predictors of overall survival for non-small cell lung cancer in never smokers. Cancer Res.

[5] Brothers JF, Hijazi K, Mascaux C, El-Zein RA, Spitz MR, Spira A (2013). Bridging the clinical gaps: genetic, epigenetic and transcriptomic biomarkers for the early detection of lung cancer in the post-National Lung Screening Trial era. BMC Med.

[6] Esteller M (2007). Cancer epigenomics: DNA methylomes and histone-modification maps. Nat Rev Genet.

[7] Risch A, Plass C (2008). Lung cancer epigenetics and genetics. Int J Cancer.

[8] Feil R, Fraga MF (2012). Epigenetics and the environment: emerging patterns and implications. Nat Rev Genet.

[9] Rodríguez-Paredes M, Esteller M (2011). Cancer epigenetics reaches mainstream oncology. Nat Med.

[10] Søes S, Daugaard IL, Sørensen BS, Carus A, Mattheisen M, Alsner J, Overgaard J, Hager H, Hansen LL, Kristensen LS (2014). Hypomethylation and increased expression of the putative oncogene ELMO3 are associated with lung cancer development and metastases formation. Oncoscience.

[11] Lin YC, Lee YC, Li LH, Cheng CJ, Yang RB (2014). Tumor suppressor SCUBE2 inhibits breast-cancer cell migration and invasion through the reversal of epithelial-mesenchymal transition. J Cell Sci.

[12] Liu WB, Jiang X, Han F, Li YH, Chen HQ, Liu Y, Cao J, Liu JY (2013). LHX6 acts as a novel potential tumour suppressor with epigenetic inactivation in lung cancer. Cell Death Dis.

[13] Liu WB, Han F, Du XH, Jiang X, Li YH, Liu Y, Chen HQ, Ao L, Cui ZH, Cao J, Liu JY (2014). Epigenetic silencing of Aristaless-like homeobox-4, a potential tumor suppressor gene associated with lung cancer. Int J Cancer.

[14] Fatemi M, Paul TA, Brodeur GM, Shokrani B, Brim H, Ashktorab H (2014). Epigenetic silencing of CHD5, a novel tumor-suppressor gene, occurs in early colorectal cancer stages. Cancer.

[15] Lee CH, Wong TS, Chan JY, Lu SC, Lin P, Cheng AJ, Chen YJ, Chang JS, Hsiao SH, Leu YW, Li CI, Hsiao JR, Chang JY (2013). Epigenetic regulation of the X-linked tumour suppressors BEX1 and LDOC1 in oral squamous cell carcinoma. J Pathol.

[16] Zhu H, Wu K, Yan W, Hu L, Yuan J, Dong Y, Li Y, Jing K, Yang Y, Guo M (2013). Epigenetic silencing of DACH1 induces loss of transforming growth factor-β1 antiproliferative response in human hepatocellular carcinoma. Hepatology.

[17] Jia Y, Yang Y, Brock MV, Zhan Q, Herman JG, Guo M (2013). Epigenetic Regulation of DACT2, A key component of the Wnt signaling pathway in human lung cancer. J Pathol.

[18] Xu L, Li X, Chu ES, Zhao G, Go MY, Tao Q, Jin H, Zeng Z, Sung JJ, Yu J (2012). Epigenetic inactivation of BCL6B, a novel functional tumour suppressor for gastric cancer, is associated with poor survival of gastric cancer. Gut.

[19] Li L, Ying J, Tong X, Zhong L, Su X, Xiang T, Shu X, Rong R, Xiong L, Li H, Chan AT, Ambinder RF, Guo Y (2014). Epigenetic identification of receptor tyrosine kinase-like orphan receptor 2 as a functional tumor suppressor inhibiting β-catenin and AKT signaling but frequently methylated in common carcinomas. Cell Mol Life Sci.

[20] Liu JY, An Q, Zhang JJ, Lei WD, Cheng SJ, Gao YN (2004). Screening of hypermethylated DNA fragments in tumor tissue derived from patients with lung cancer. Yi Chuan Xue Bao.

[21] Liu WB, Liu JY, Ao L, Zhou ZY, Zhou YH, Cui ZH, Yang H, Cao J (2009). Dynamic changes in DNA methylation during multistep rat lung carcinogenesis induced by 3-methylcholanthrene and diethylnitrosamine. Toxicol Lett.

[22] Liu WB, Liu JY, Ao L, Zhou ZY, Zhou YH, Cui ZH, Cao J (2010). Epigenetic silencing of cell cycle regulatory genes during 3-methylcholanthrene and diethylnitrosamine induced multistep rat lung cancer. Mol Carcinog.

[23] Liu WB, Ao L, Zhou ZY, Cui ZH, Zhou YH, Yuan XY, Xiang YL, Cao J, Liu JY (2010). CpG island hypermethylation of multiple tumor suppressor genes associated with loss of their protein expression during rat lung carcinogenesis induced by 3-methylcholanthrene and diethylnitrosamine. Biochem Biophys Res Commun.

[24] Liu WB, Cui ZH, Ao L, Zhou ZY, Zhou YH, Yuan XY, Xiang YL, Liu JY, Cao J (2011). Aberrant methylation accounts for cell adhesion-related gene silencing during 3-methylcholanthrene and diethylnitrosamine induced multistep rat lung carcinogenesis associated with overexpression of DNA methyltransferases 1 and 3a. Toxicol Appl Pharmacol.

[25] Liu WB, Ao L, Cui ZH, Zhou ZY, Zhou YH, Yuan XY, Xiang YL, Cao J, Liu JY (2011). Molecular analysis of DNA repair gene methylation and protein expression during chemical-induced rat lung carcinogenesis. Biochem Biophys Res Commun.

[26] Liu WB, Han F, Jiang X, Yang LJ, Li YH, Liu Y, Chen HQ, Ao L, Cui ZH, Cao J, Liu JY (2012). ANKRD18A as a novel epigenetic regulation gene in lung cancer. Biochem Biophys Res Commun.

[27] Liu WB, Han F, Jiang X, Yin L, Chen HQ, Li YH, Liu Y, Cao J, Liu JY (2015). Epigenetic regulation of ANKRD18B in lung cancer. Mol Carcinog.

[28] Hwang SJ, Yang Q, Meigs JB, Pearce EN, Fox CS (2007). A genome-wide association for kidney function and endocrine-related traits in the NHLBI's Framingham Heart Study. BMC Med Genet.

[29] Fry JL, Toker A (2010). Secreted and membrane-bound isoforms of protease ADAM9 have opposing effects on breast cancer cell migration. Cancer Res.

[30] Wang L, Jin Y, Arnoldussen YJ, Jonson I, Qu S, Maelandsmo GM, Kristian A, Risberg B, Waehre H, Danielsen HE, Saatcioglu F (2010). STAMP1 is both a proliferative and an antiapoptotic factor in prostate cancer. Cancer Res.

[31] Anami K, Oue N, Noguchi T, Sakamoto N, Sentani K, Hayashi T, Hinoi T, Okajima M, Graff JM, Yasui W (2010). Search for transmembrane protein in gastric cancer by the Escherichia coli ampicillin secretion trap: expression of DSC2 in gastric cancer with intestinal phenotype. J Pathol.

[32] Yu F, Ng SS, Chow BK, Sze J, Lu G, Poon WS, Kung HF, Lin MC (2011). Knockdown of interferon-induced transmembrane protein 1 (IFITM1) inhibits proliferation, migration, and invasion of glioma cells. J Neurooncol.

[33] Young J, Biden KG, Simms LA, Huggard P, Karamatic R, Eyre HJ, Sutherland GR, Herath N, Barker M, Anderson GJ, Fitzpatrick DR, Ramm GA, Jass JR (2001). HPP1: a transmembrane protein-encoding gene commonly methylated in colorectal polyps and cancers. Proc Natl Acad Sci USA.

[34] Guo X, Jing C, Li L, Zhang L, Shi Y, Wang J, Liu J, Li C (2011). Down-regulation of VEZT gene expression in human gastric cancer involves promoter methylation and miR-43c. Biochem Biophys Res Commun.

[35] Scott R, Siegrist F, Foser S, Certa U (2011). Interferon-alpha induces reversible DNA demethylation of the interferon-induced transmembrane protein-3 core promoter in human melanoma cells. J Interferon Cytokine Res.

[36] Duvvuri U, Shiwarski DJ, Xiao D, Bertrand C, Huang X, Edinger RS, Rock JR, Harfe BD, Henson BJ, Kunzelmann K, Schreiber R, Seethala RS, Egloff AM (2012). TMEM16A induces MAPK and contributes directly to tumorigenesis and cancer progression. Cancer Res.

[37] Akaishi J, Onda M, Okamoto J, Miyamoto S, Nagahama M, Ito K, Yoshida A, Shimizu K (2007). Down-regulation of an inhibitor of cell growth, transmembrane protein 34 (TMEM34), in anaplastic thyroid cancer. J Cancer Res Clin Oncol.

[38] Rosemond E, Rossi M, McMillin SM, Scarselli M, Donaldson JG, Wess J (2011). Regulation of M3 muscarinic receptor expression and function by transmembrane protein 147. Mol Pharmacol.

[39] Wang L, Yu C, Lu Y, He P, Guo J, Zhang C, Song Q, Ma D, Shi T, Chen Y (2007). TMEM166, a novel transmembrane protein, regulates cell autophagy and apoptosis. Apoptosis.

[40] Chang Y, Li Y, Hu J, Guo J, Xu D, Xie H, Lv X, Shi T, Chen Y (2013). Adenovirus vector-mediated expression of TMEM166 inhibits human cancer cell growth by autophagy and apoptosis in vitro and in vivo. Cancer Lett.

[41] Li L, Khatibi NH, Hu Q, Yan J, Chen C, Han J, Ma D, Chen Y, Zhou C (2012). Transmembrane protein 166 regulates autophagic and apoptotic activities following focal cerebral ischemic injury in rats. Exp Neurol.

[42] Takeuchi T, Adachi Y, Nagayama T (2012). A WWOX-binding molecule, transmembrane protein 207, is related to the invasiveness of gastric signet-ring cell carcinoma. Carcinogenesis.

[43] Li C, Wei J, Li Y, He X, Zhou Q, Yan J, Zhang J, Liu Y, Liu Y, Shu HB (2013). Transmembrane protein 214 (TMEM214) mediates endoplasmic reticulum stress-induced caspase-4 activation and apoptosis. J Biol Chem.

