# Supplementary material for: TMEM196 acts as a novel functional tumour suppressor inactivated by DNA methylation and is a potential prognostic biomarker in lung cancer
Source: Oncotarget. 2015 May 22;6(25):21225–39. doi: 10.18632/oncotarget.4237 (PMC4673261; doi:10.18632/oncotarget.4237)
Supplement: Supplementary file 1 [file oncotarget-06-21225-s001.pdf]

TMEM196 acts as a novel functional tumour suppressor inactivated by DNA methylation and is a potential prognostic biomarker in lung cancer

Supplementary Material

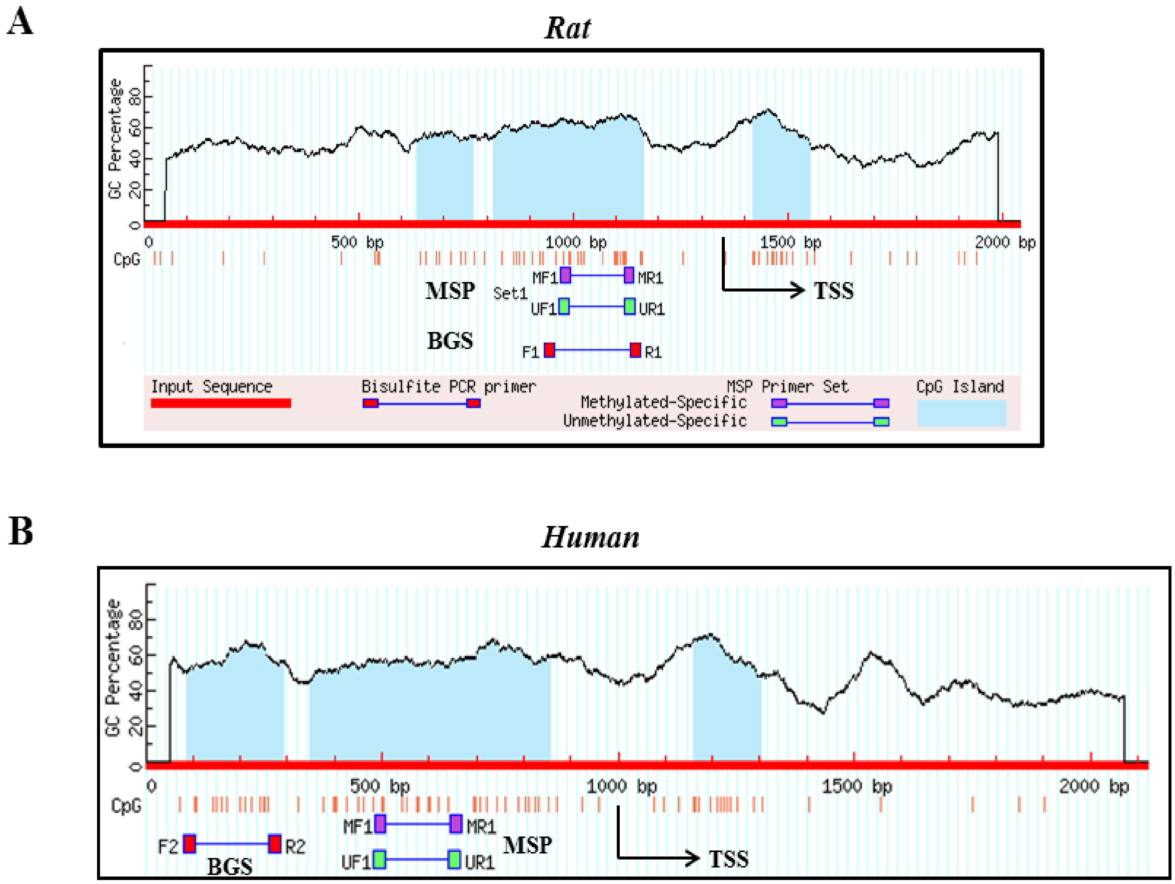

Supplementary Figure S1: Schematic for the location of the CpG islands in the MSP region and BGS region in the transcription start region of rat (A) and human (B) *TMEM196* gene. TSS: transcription start site.

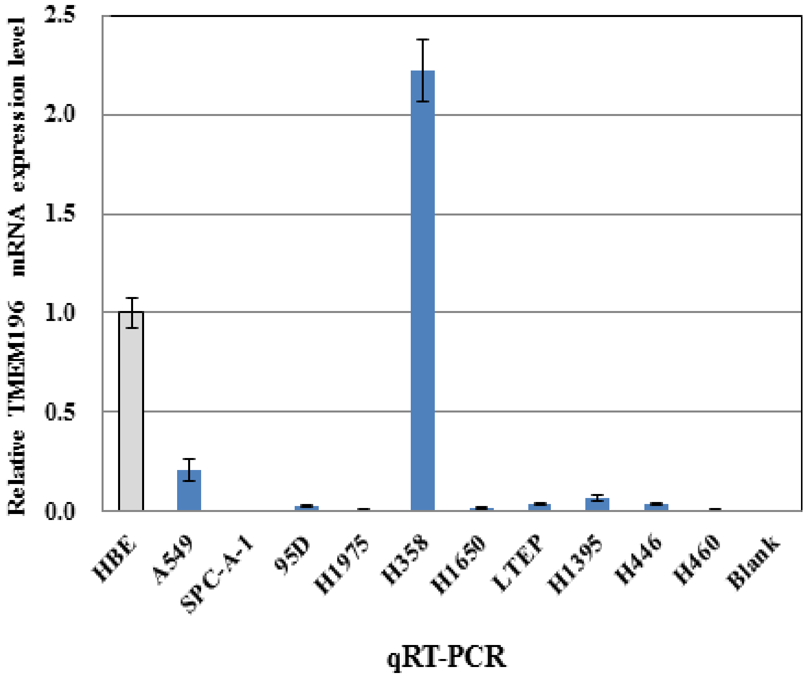

Supplementary Figure S2: Expression of *TMEM196* was analysed by quantitative RT-PCR in lung cancer cell lines and HBE cells. *TMEM196* was downregulated or silenced in all human lung cancer cell lines, except for H358, compared with HBE normal cells.

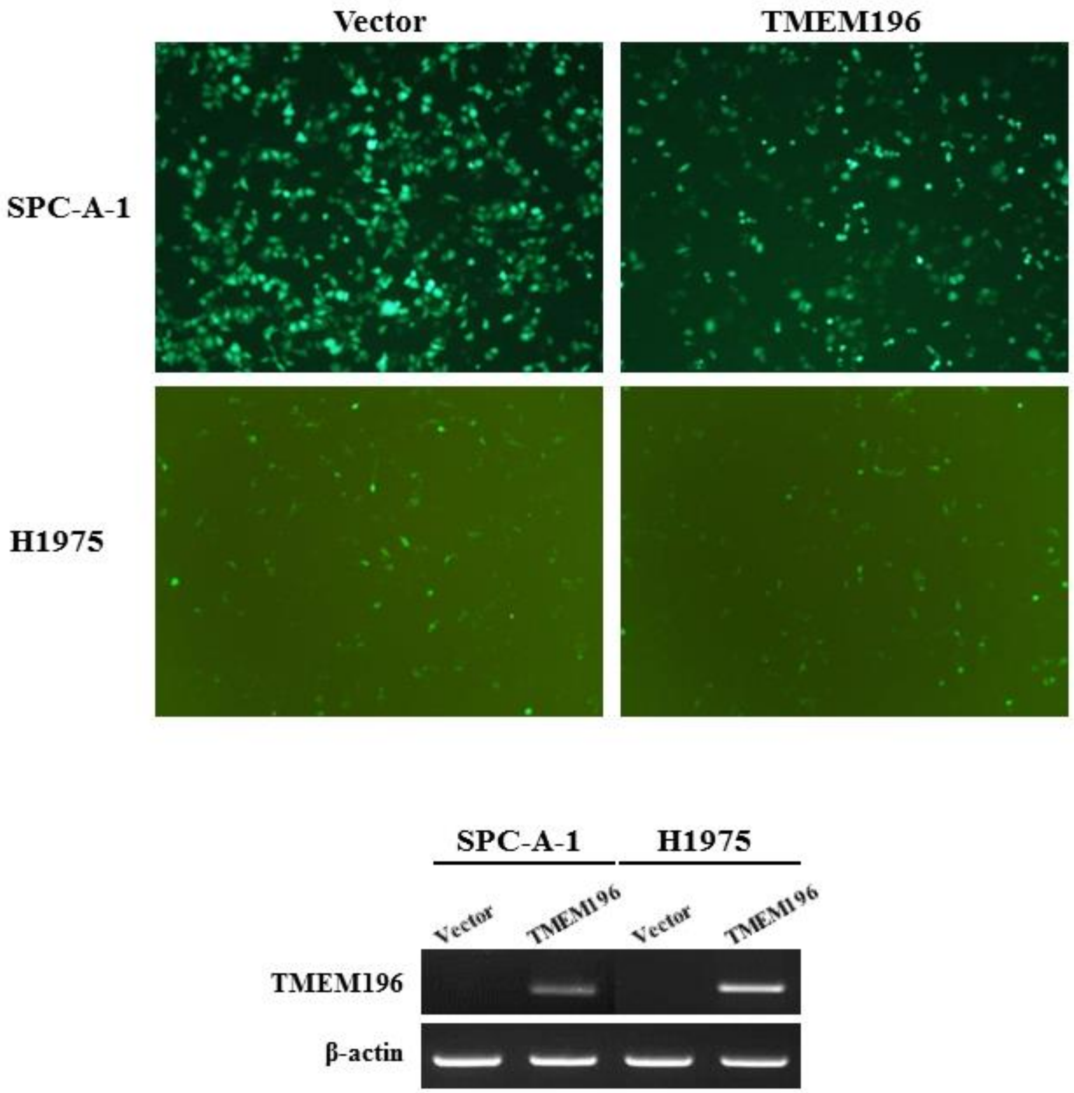

Supplementary Figure S3: Transfection efficiency was evaluated in SPC-A-1 and H1975 cells transfected with pIRES2-EGFP for 48 h by EGFP observation under a fluorescence microscope and RT-PCR analysis.

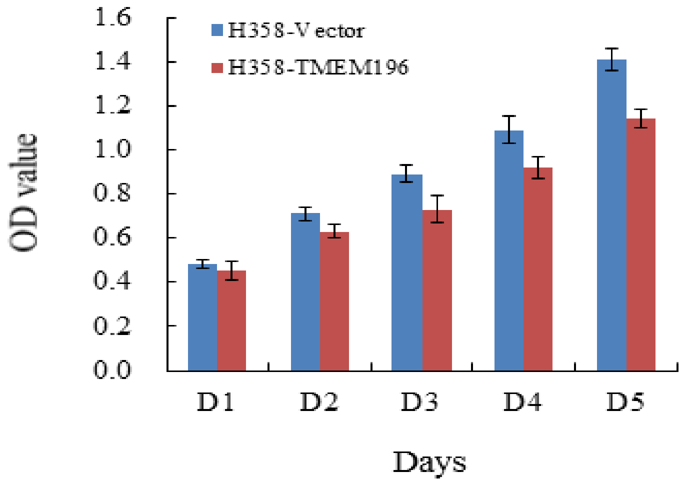

Supplementary Figure S4: *TMEM196* overexpression inhibited cancer cell growth in H358 cells with normal *TMEM196* expression by CCK-8 assay.

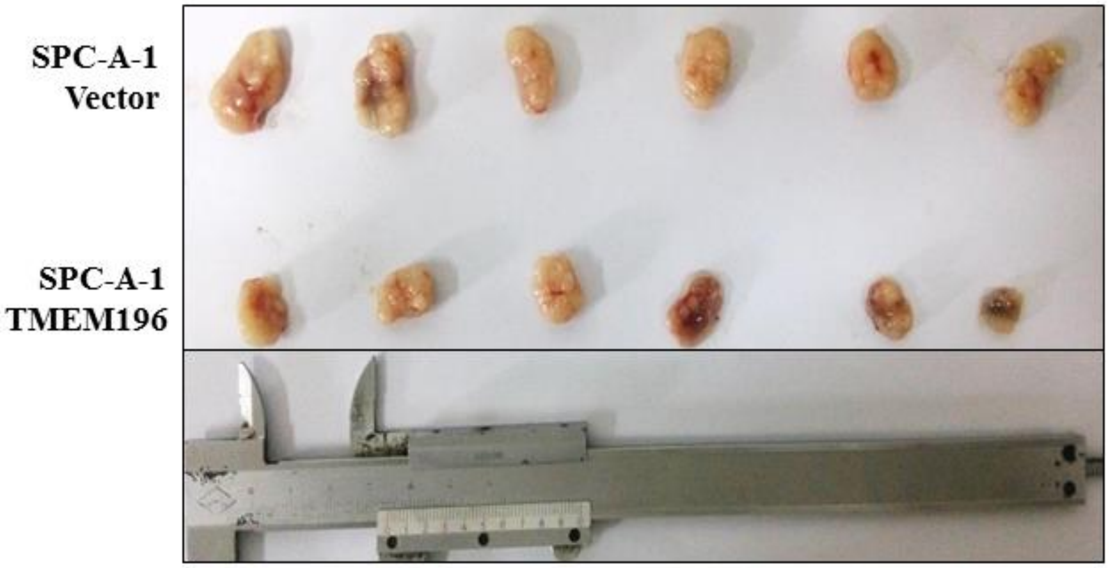

Supplementary Figure S5: Pictures of the isolated tumours excised 3 weeks after injection of stably transfected cells into nude mice (n = 6).

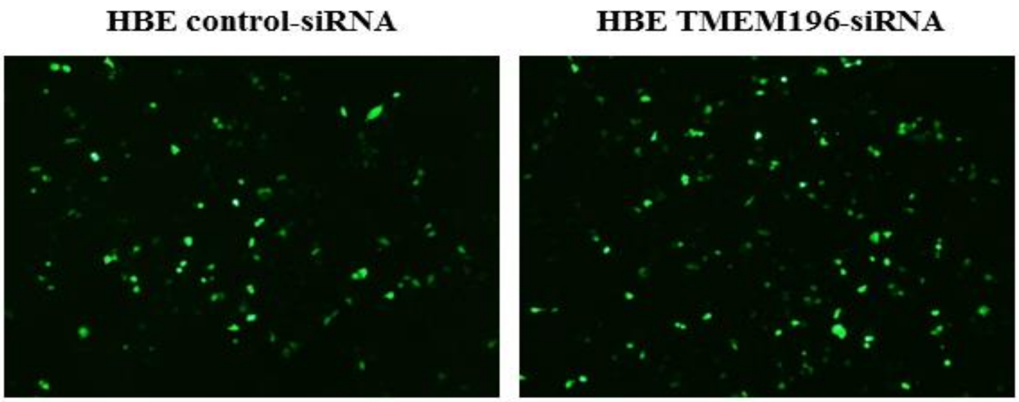

Supplementary Figure S6: Knockdown efficiency by siRNA was evaluated in HBE cells at 48 h after transfection by EGFP observation under a fluorescence microscope.

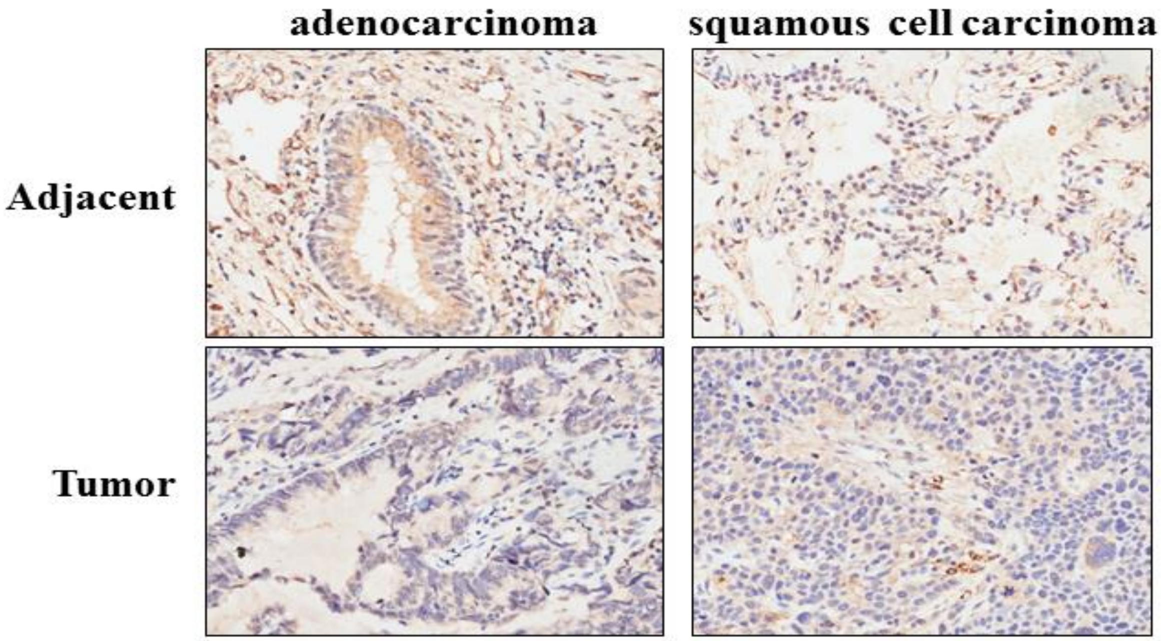

Supplementary Figure S7: Representative images of IHC staining showed that TMEM196 was expressed at low levels in tumour tissues and highly expressed in adjacent tissue samples of lung adenocarcinoma and squamous cell carcinoma.

Supplementary Table S1: Primer sequences used in this study

Gene		Primer sequence(5'-3')	Length(bp), position	Annealing temperature(℃)
Rat-*TMEM196*	MSP(M)	Forward: GTTTTTGGAAGCGTAGAGTAGAGAC	174,	56
		Reverse: AAAACTAATCCAACCCGTAACG	-376 to -203	
	MSP(U)	Forward: GGTTTTTGGAAGTGTAGAGTAGAGAT	178,	54
		Reverse: AACAAAACTAATCCAACCCATAACA	-377 to -200	
	BGS	Forward: GTTAGGAAGGAGAAGGGAGTTAGAG	220,	56
		Reverse: CAAAAAAAACAAAACTAATCCAACC	-412 to -193	
Human-*TMEM196*	MSP(M)	Forward: TGGTGTTGAATATTATTCGTTCGT	184,	54
		Reverse: CAAAATCCCTTCTATTTTATTACCG	-516 to -333	
	MSP(U)	Forward: GTGGTGTTGAATATTATTTGTTTGT	183,	52
		Reverse: AAATCCCTTCTATTTTATTACCAAA	-517 to -335	
	BGS	Forward: TTTTTGTTTATTGTTTTTTTTATTT	207,	52
		Reverse: CAATACTCTTAACTAACCTTTAATCC	-921 to -715	
	RT-PCR	Forward: GTGCTGGAGATAGGGC	265,	52
		Reverse: GCAGTGGGTATAGGGA	131 to 395	
	qRT-PCR	Forward: CATTGAACCACATGCTGGAA	134,	60
		Reverse: ATGGTCCAAAGTATACCACCACTC	3030 to 3163	

Supplementary Table S2: *TMEM196* methylation and protein expression in the chemical-induced, rat lung carcinogenesis model

Pathological status	Numbers	Methylation			Expression		
		M	U	Frequency (%)	+	−	Positive rate (%)
Normal	20	0	20	0.0	20	0	100.0
Hyperplasia	25	0	25	0.0	22	3	88.0
Squamous metaplasia	27	4	23	14.8	21	6	77.8
Dysplasia	37	11	26	29.7 [a, c]	22	15	59.5 [b, d]
CIS	30	12	18	40.0 [b, c, e]	16	14	53.3 [b, c]
Infiltrating carcinoma	25	13	12	52.0 [b, c, f]	11	14	44.0 [b, c, e]

U: unmethylated; M: methylated.

[a] Normal *versus* dysplasia, $P < 0.05$;

[b] Normal *versus* dysplasia, CIS or infiltrating carcinoma, $P < 0.01$;

[c] Hyperplasia *versus* dysplasia, CIS or infiltrating carcinoma, $P < 0.01$;

[d] Hyperplasia *versus* dysplasia, $P < 0.05$;

[e] Squamous metaplasia *versus* CIS or infiltrating carcinoma, $P < 0.05$;

[f] Squamous metaplasia *versus* infiltrating carcinoma, $P < 0.01$;

Supplementary Table S3: Clinical factor and *TMEM196* methylation in 62 cases of lung cancer

Clinical factor	Numbers	*TMEM196* methylation status		Methylated(%)	*P*
		Unmethylated	Methylated		
Age(year)					0.080
<60	21	12	9	42.9	
>60	41	14	27	65.9	
Gender					0.670
male	47	19	28	59.6	
female	15	7	8	53.3	
Smoking					0.098
Yes	45	16	29	64.4	
No	17	10	7	41.2	
Differentiation					0.039
Poor	6	2	4	66.7	
Moderate	42	14	28	66.7	
Well	14	10	4	28.6	
Pathological type					0.378
SCC	28	14	14	50	
ADC	21	7	14	66.7	
ASC	7	3	4	57.1	
SCLC	5	1	4	80	
LCLC	1	1	0	0	
Stage					0.017
I and II	45	23	22	48.9	
III and IV	17	3	14	82.4	

Supplementary Table S4: Correlation of TMEM196 expression and *TMEM196* hypermethylation in the chemical-induced, rat lung carcinogenesis model

Pathological status	Methylated *TMEM196*		Unmethylated *TMEM196*	
	TMEM196 positive	TMEM196 negative	TMEM196 positive	TMEM196 negative
Normal	0	0	20	0
Hyperplasia	0	0	22	3
Squamous metaplasia	0	4	21 [a]	2
Dysplasia	0	11	22 [a]	4
CIS	0	12	16 [a]	2
Infiltrating carcinoma	0	13	11 [a]	1
Total	0	40	112 [a]	12

[a] $P < 0.01$
